# Current knowledge, attitudes and practices of expectant women toward routine sonography in pregnancy at Naguru health centre, Uganda

**Published:** 2009-11-30

**Authors:** Mubuuke Aloysius Gonzaga, Elsie Kiguli-Malwadde, Businge Francis, Byanyima Rosemary

**Affiliations:** 1 Makerere University, College of Health Sciences, Radiology Department,; 2 Mulago Hospital, Radiology Department

**Keywords:** Pregnancy, Routine sonography, Naguru health centre, Uganda

## Abstract

**Background:**

Ultrasound has become a routine part of care for pregnant women in Uganda, being one of a range of techniques used in screening. However, it differs from most others because it allows women to view their babies. Routine obstetric sonography is now globally recognized as one of the ways through which maternal mortality can be reduced. This study aimed at finding out the knowledge, attitudes and practices of pregnant women towards prenatal sonography at Naguru Health Centre, Uganda.

**Method:**

Exploratory -descriptive study using interviewer-administered questionnaires. Thematic analysis was employed for qualitative data and bivariate, multivariate and logistic regression analysis was used for quantitative data.

**Results:**

Three themes emerged; Knowledge, Attitude and Practices. Women’s knowledge, attitude and practices of obstetric sonography were influenced mainly by their biosocial factors like gravidity, education level and occupation. All women expressed concern that obstetric sonography could lead to cancer.

**Conclusion:**

Obstetric sonography is highly appreciated as being vital for antenatal care. However, there is need for mothers and health care providers to be well informed about the safety and specific purposes of obstetric sonography and what it can and cannot achieve.

## Background

There has been increased medicalization of pregnancy globally due to advances in technology in the field of healthcare and most especially in obstetric care [1]. The predominance of this perception is rooted in a number of trends extending over a period of time. In support of these trends, policy makers cite the reduction in maternal and perinatal morbidity and mortality as justification for all the changes made in obstetrical care [2,3]. Routine obstetric ultrasound has been one of the most important advances in antenatal care worldwide [2–5].

However, it has been reported that innovative medical technologies like obstetric sonography have the potential to raise social, ethical and economic dilemmas for both health workers and the recipients of health services [5]. Uganda is a land locked country in Eastern Africa with a total land area of 236040 square kilometers. It has a total population of 28.3 million, 87.7% of whom live in rural areas and have limited access to tertiary education [6]. In Uganda, health care delivery has been decentralized from the top to the bottom with the aim of bringing services nearer to the people. From the top downwards, there are National referral Hospitals, Regional referral Hospitals, District Hospitals and Health Centre IV to I at County, Sub-county, Parish and Village levels, respectively [7]. In most of these health care facilities including private health care facilities, routine obstetric sonography has been fully embraced.

While obstetric sonography has proven to be beneficial in situations where it is indicated, the role of it being routine remains contentious [8,9]. There is a wealth of literature about the psychosocial effects and therapeutic benefits of prenatal sonography. Bashour et al and Georgsson et al reported that the ultrasound experience will reassure the pregnant woman about fetal well-being, encourage women to abandon practices harmful to the fetus, facilitate early bonding and will be enjoyable and interesting [9,10]. Indeed many women have got many expectations from routine scans like knowing the fetal sex, status of the baby and expected date of delivery [11,12]. Whynes further reports that, most women now accept the scan uncritically because of the enormous expectations they have, but most especially viewing their babies live on the screen and knowing the fetal sex [13]. Nigenda et al concurred with the aforementioned views when they reported that pregnant women attending antenatal care in developing countries have got several expectations when they are sent for ultrasound, most which are about knowing the sex of the fetus, viability, expected due date and the reassurance that the baby is fine [14].

Conversely, Tautz et al advise that sometimes pregnant women have got over expectations that may not be met during scanning which creates a different feeling for them after the scan [8]. This is mostly encountered with women who have some knowledge about obstetric sonography. It has been reported that women with higher levels of formal education are more likely to have many expectations as well as ask many questions compared to women with low levels of formal education [15]. The health care providers have also contributed in a way to this obsession. Gammeltoft and Nguyen report that health workers themselves have declared obstetric ultrasound an indispensable part of modern antenatal care and therefore recommend it. This has created a dramatic overuse of this technology mainly because of its over commercialization for monetary gains in both public and private health facilities. For example in a survey carried out in Viet Nam, 400 women had an average of 6.6 scans during their pregnancy and one-fifth had had ten scans or more [5]. Gammeltoft and Nguyen conclude by suggesting the need for guidelines for the appropriate use of obstetric ultrasound in antenatal care. Similar findings and suggestions were reported by Bashour et al in their study with Syrian women [9]. It has been reported that majority of women especially in developed countries no longer have fears regarding the safety of ultrasound, and so go for it uncritically [16].

The paucity of literature in the Ugandan situation exploring this issue warrants attention. By listening to women talk, health workers and policy makers may have an enhanced and broad understanding of the knowledge, attitudes and perceptions these women have about obstetric ultrasound. The purpose of the present study was to explore knowledge, attitudes and perceptions of pregnant women about obstetric scanning, whose findings are documented below.

## Method

### Study site

The study was conducted at Naguru Health Centre IV, Kampala district, Uganda. Naguru Health Centre offers out-patient services, maternity, immunization, general theatre services with a capacity of about 25 beds and 35 full time staff.

### Study design

It was an exploratory study in which semi-structured key-informant individual interviews were conducted. Individual interviews help to collect insightful descriptions from participants [17,18]. Open-ended questions were used and responses tape-recorded. Each interview began with obtaining consent from the participant, explanation of the study followed by a discussion of any concerns. Participants were assured of the confidentiality of their opinions. The tape-recorded interview followed the completion of the aforementioned tasks. Demographic information for each participant was also collected. All the participants of this study had had an ultrasound in their pregnancy. There were no restrictions to maternal age and gestational week of the pregnancy. Primigravida and multiparous women were all included to obtain feelings and attitudes of both groups. There were, however restrictions to women having any complication(s) as the pregnancy experience may be extremely different for them. In order to ensure validity, all the researchers participated in data collection to ensure triangulation by having a team research approach. At the same time triangulation was done by comparing data to already existing literature, and transcripts were always kept and referred to during the research period and the participants were requested to verify the recorded interview on the tape; all to ensure validity.

### Sample size

Thirty women were considered for the study through convenience sampling. This sample size provided enough data saturation whereby the responses had become repetitive and redundant at the 30th woman and no new ideas were coming up. At the same time, the responses got provided enough depth which is key in focused ethnography exploratory qualitative studies like this study rather than dwelling on the breadth of the sample size that provide similar responses even after data saturation [19].

### Data analysis

Thematic content analysis, a valued method for analyzing qualitative data was used [20]. This involved content analysis to extract the meanings of the informants, and also transcription. Raw data was proof-read against audio-taped interviews and coded into categories of similar meaning. This technique is one Wilson collectively refers to as analytic description [21]. Categories were established, resulting into content themes, consistent with the value of thematic content analysis in qualitative methods [22]. These themes summarized the meaning of the data which addressed the purpose of the study. Bivariate, multivariate and logistic regression model were the methods used to analyse the quantitative part of the data using SPSS. A Chi-square was used to test statistical significance and a p-value of 0.05 was adopted for this study.

### Ethical issues

Permission was obtained from the Radiology department of Makerere University and also from Naguru Health Centre. Consent was also obtained from each study participant. Confidentiality, autonomy, respects and dignity of all the participants was strictly observed throughout the study. Additionally participants were assured of their rights to decline participating in the study and also not to answer questions they felt uncomfortable with. The participants were also assured that there will be no harm, prejudice, malice or any form of danger should they wish not to participate in the study. This study strictly adhered to the principles of the Declaration of Helsinki.

## Results

### Demographic Information

Thirty women participated in this study with age range of 19 to 42 years, and an average age of 28.3 years. 66.7% (n=20) were Christians and 33.3% (n=10) were Muslims. 43.3% (n=13) were primigravida and 56.7% (n=17) had been pregnant before. All were pregnant at the time of their interview. Of the 30 women interviewed, 16.7% (n=5) were in the 10 ^th^ gestation week, 13.3% (n=4) were in the 17 ^th^ gestation week, 10% (n=3) were in the 23 ^rd^ gestation week, 6.7% (n=2) were in the 28 ^th^ gestation week, 10% (n=3) were in the 32 ^nd^ gestation week, 10% (n=3) were in the 33 ^rd^ gestation week and the remaining 33.3% (n=10) were above the 33 ^rd^ week of gestation. 16.7% (n=5) were undergoing scan for the first time, 66.6% (n=20) were doing scan for the second time while the remaining 16.7% (n=5) were doing it for the third time. All had partners at the time of the interview. 26.7% (n=8) of the women had no formal education, 33.3% (n=10) had attained primary level education, 23.3% (n=7) had secondary education and 16.7% (n=5) had tertiary level education. 26.7% (n=8) were not employed, 33.3% (n=10) were market vendors, 23.3% (n=7) were retail shopkeepers, 6.6% (n=2) were nurses, 6.6% (n=2) were primary school teachers and 3.5% (n=1) was a secondary school teacher.

### Themes

Three key themes were identified: knowledge, attitude and practices.

### Knowledge

All women reported having some knowledge about obstetric sonography no matter the level of education, parity or type of occupation. However, knowledge levels varied depending on the level of education. For example the 2 nurses in this study cited the uses of obstetric sonography which included determining fetal presentation and lie, expected date of delivery, location of placenta, checking whether the cord was around the neck and assessing the fetal parts. On the contrary, the 8 women without formal education knew the existence of obstetric scan for showing that the baby is alive as one lady explicitly put it in a local language because she could not speak English: “Ka T.V kakeebera ob`omwana mulamu kyoka, ebirara tekasobola kubiraba”, literally meaning that ultrasound simply checks for fetal viability. Pressed further, they could not give any more reasons. The women with primary education cited other reasons like assessing fetal movements and number of fetuses. The secondary school teacher went ahead to cite reasons like checking any abnormalities of the mother’s organs and uterus in addition to checking the baby’s viability.

The source of knowledge again varied according to level of education. The nurses, primary and secondary school teachers cited sources like radio and T.V programmes, newspapers, health promotion activities and peers, while the market vendors cited talks given by mid-wives when they go antenatal check-ups and their peers only. However, all the 30 women in this study expressed that they know ultrasound may lead to cancer, regardless of their level of education. When asked about the source of this knowledge, it was a common thread that they had got it from their friends.

“My friend told me that over-getting exposed to the scan may shorten my life through acquisition of cancer for me and my baby”, one secondary school teacher said.

On further exploration, all the women expressed willingness to learn more about routine obstetric scan and whether it has any effects to their lives and that of their babies. They also expressed deficit in knowledge about what ultrasound can and cannot do.

### Attitude

All the women reported a positive attitude towards obstetric sonography with 66.7% (n=20) of them saying that the scan could help them plan better for their pregnancies. 83.3% (n=25) had a positive feeling about scanning and accepted it uncritically when their doctor requested for it.

“I even asked my doctor to request for me a scan just to see my baby jumping on the T.V”, one lady asserted. “It feels good to know the sex of the baby in advance and I start preparing for him or her. This makes me enjoy my pregnancy more”, another lady said.

However, all the women expressed their feelings about the safety of their lives and the lives of their babies due to overexposure to ultrasound.

“Sometimes I get worried that I may get cancer due to this scan because my friends have told me so”, one lady said.

The fear of getting cancer due to exposure to the scan was a common perception in all the responses. Additionally, all women expressed their dissatisfaction with the person doing the scan due to lack of proper communication with them. Many of them had questions, but were not either responded to or they were responded to rudely as one lady put it,

“When the doctor shouted at me for asking many questions, I lost all the excitement I had of seeing my baby on the T.V”.

### Practices

All women reported that they duly accepted to go for the scan in their pregnancy. Some even reported requesting for it themselves. The commonest reasons cited for them to willingly go the scan were to know the fetal sex (100%), expected date of delivery (100%) and viability (81.2%). One lady, pregnant for the second time, described her need for the scan:

“I wanted it to be routine and that's why I asked the doctor to order for me one even if I was feeling fine”.

73.3% (n=22) women wanted a scan for “reassurance” that the baby was fine. All the 30 women said that they would actually go for a scan even if their doctor did not request for it just to look at the baby.

Five women expressed dissatisfaction however, about the false information got in their previous scans as one of them explicitly put it:

“I was told when I went to the scan that I had a baby boy. I did all my shopping buying only blue things, only to give birth to a girl. I no longer have trust in those scan things and I may not go for it again”.

Regarding knowing fetal viability, factors like age, occupation and education level significantly influenced the decision to seek for the scan on bivariate analysis (p=0.004), but all were not significant on multivariate analysis (p=0.094).

Regarding knowing fetal sex as a driving force to seek for the scan; age, religion, occupation and gravidity were significant on bivariate analysis (p=0.002), however only gravidity and level of education remained the significant independent variables on logistic regression model (p=0.003). Primigravida women were about four times more likely to request for the sex of the baby than multi-parous women, (OR 3.78, 95% CI 1.51–9.43). Women with some formal education were seven times more likely than the uneducated ones to request for the sex of the fetus, (OR 6.9, 95% CI 1.46–333.21). The impendiment to do routine obstetric scans expressed by all women was the fear for its effects to their lives and their babies; most especially the fear to get cancer from exposure to ultrasound was expressed by all women.

## Discussion

### Knowledge

This study involved women with a range of biosocial variables. Regarding their knowledge about the use of obstetric sonography, all women expressed some level of knowledge about obstetric sonography. This is partly due to the wide use of ultrasound in health care today as part of routine antenatal care as well as the unlimited access to information. However, the kind of knowledge these women have again varies mainly due to their level of education.

The level of education tends to influence the methods in which these women acquire and synthesize information about ultrasound. Women with lower levels of formal education tend to be limited to only peers and when they go for antenatal checkups which expose them to limited information about obstetric sonography. This is why it noted that such women think that the scan is to only check whether the baby is alive or not. On the contrary, women with higher levels of formal education had diverse information sources like newspapers, radio programmes. This is partly because they can read, listen and comprehend this information, being literate. Therefore these women could cite very many other pertinent indications for obstetric scan. This therefore shows the significance of some level of formal education in society as it equips people with skills to access reliable information.

All women in this study said they know that ultrasound can lead to cancer. This is misleading information got from friends and peers which is not justified. This is a critical point this study has brought out. Women in the developed world uncritically go for obstetric scan without any of the fears expressed in this study [13]. However, this is a different issue in Uganda and probably in the rest of the developing world. Health care providers need to come out and address the issues raised so as to cover the knowledge gap of the public about the safety of ultrasound.

### Attitude

All women reported having a positive attitude towards obstetric sonography. This is partly due to the excitement these women have in anticipation of seeing their babies on the scan. Probably seeing their babies playing around, knowing the fetal sex, knowing the expected date of delivery and just knowing that their baby was fine are very important factors that influence the women’s perception of obstetric sonography. It allows them to see the progress of their babies which creates an early fetal-maternal bonding even before birth. So this makes obstetric sonography popular and highly regarded by pregnant women.

Indeed, many pregnant women are bound to make self-requests for ultrasound even when it is not indicated by the doctor just to see their babies or to know the sex of the baby. Healthcare providers need to be aware of this. The fear of cancer again has influenced women’s perception of ultrasound, and a number of them are worried about this. This is a serious issue that needs to be addressed by healthcare providers. An important issue of health workers not responding to patients during the scan is of great concern. This has influenced the way patients perceive these health workers. This is due to poor interpersonal skills demonstrated by the people doing the scan and this trickles down to their training which must change, so that health workers communicate with patients effectively. The introduction of Problem Based Learning/Community Based Education and Service at the College of Health Sciences, Makerere University was aimed at producing graduates with good interpersonal to address this issue [23]. The importance of communication with patients during the scan was also highlighted by Whynes [13].

### Practices

All women reported accepting doing a scan as requested by the health workers. The compliance of women to do the scan may be explained by the perceived benefits these women expect to get from the scan, some of which include knowing fetal sex, expected due date and fetal viability. Knowing that the scan can give information on all these is possibly the major reason as to why women readily go for it even when their doctor has not requested for it. However, the urge to do an obstetric scan is probably influenced by other biosocial factors as this study has shown. For example, primigravid women were more likely to go for a scan simply to know the sex of the baby as opposed to multi-parous women. This may be due to excitement of being a mother for the first time and preparing for the baby in advance, an experience that raises high hopes in women of first time pregnancies. This finding is similar to what Enakpene et al reported [15].

However, careful consideration should be taken when telling women the sex of their babies. This raises numerous ethical, legal and social dilemmas, in addition to how this knowledge of fetal sex affects a woman’s emotions and relationship with her unborn child before and later on in life. It should be remembered that there are always false positives and false negatives with fetal sex which may have numerous implications like selective abortions of unwanted fetal genders. Williams et al also warn about the ethical implications of telling pregnant women the fetal gender [4].

Bashour et al also caution about the excessive misuse of obstetric sonography even when it is not justified due to its commercialization for monetary gains by health workers [9]. Some pregnant women are even making self-requests for the scan without any valid indication for it from a health worker just because they can pay for it. This study also uniquely brings out an important issue; women think that ultrasound can actually lead to cancer and affect them as well as their babies, and this perception runs through from the illiterate to the literate ones. This is a perception women have and which needs to be urgently addressed in Uganda and probably globally. The sample size used in this study is a major limitation; however, the findings herein are useful to all health workers and policy makers and open a way for further research in this area.

## Conclusion

Obstetric sonography has been embraced as being a vital part of pre-natal care. Most women want it and duly go for it. However, guidelines are needed for the appropriate use of ultrasound in pregnancy and how best to combine it in the antenatal health care package and not just over-commercializing it. The perception of women that sonography can lead to cancer needs to be addressed and health care providers and mothers should know the purpose of sonography in pregnancy and what it can and cannot achieve.
